# Suicide Screening in the Oncology Population

**Published:** 2016-01-01

**Authors:** Mary K. Hughes

**Affiliations:** The University of Texas MD Anderson Cancer Center, Houston, Texas

Registered nurses spend more time with patients than other health-care workers and are in a prime position to detect and prevent suicidal behaviors in patients, but Valente (2010) found that oncology nurses had more difficulty in caring for suicidal patients because of their commitment to preserving life. Although a relatively rare event, suicide increases in patients with the diagnosis of cancer (Misono, Weiss, Fann, Redman, & Yueh, 2008). Shneidman (1985) defined suicide as "the conscious act of self-induced annihilation, best understood as a multidimensional malaise in a needful individual who defines an issue for which the act is perceived as the best solution." It is not random or pointless but a release from an issue or crisis that produces intense suffering (Kaplan & Sadock, 1998).

The most common mental illness found in cancer-related suicide cases is depression (75%; de la Grandmaison, Watier, Cavard, & Charlier, 2014). The prevalence of depression among patients with cancer is between 5% and 16% (Walker et al., 2013). Since fatigue and depression occur concurrently, fatigue can be a cue for practitioners to investigate for depression (Rhondali et al., 2012). Multiple studies have identified a higher risk factor for suicide during the first months after a cancer diagnosis as well as 1 year after (Anguiano, Mayer, Piven, & Rosenstein, 2012).

## Patients at Risk 

Who is more vulnerable to suicide after a cancer diagnosis? There are four site-specific malignancies with higher suicide rates than other sites: the head and neck, lungs, pancreas, and prostate (Lydiatt, Moran, & Burke, 2009; Urban et al., 2013; Carlsson et al., 2013; Miller, Mogun, Azrael, Hempstead, & Solomon, 2008; Turaga, Malafa, Jacobsen, Schell, & Sarr, 2011). Men with testicular cancer have a 20% increase in the risk of suicide over that of the general population (Alanee & Russo, 2012). Klaassen et al. (2015) found that patients with bladder cancer posed the highest risk for suicide within the first 5 years after diagnosis. Mohammadi and colleagues (2014) found that patients with myeloma had the highest rate of attempted as well as completed suicide of those with hematologic malignancies.

Adult survivors of childhood cancers have higher suicidality (Recklitis et al., 2010). Other risk factors for suicide include being male or being over 65 (Anguiano et al., 2012). Women with gynecologic cancer, especially ovarian, have a higher incidence of suicide than women with other cancers (Ward, Roncancio, & Plaxe, 2012; Tang et al., 2015).

Clinical factors associated with suicidality include substantial pain, insomnia, fatigue, loss of autonomy and independence, poor social support, impaired physical functioning, demoralization, and emotional distress (de la Grandmaison et al., 2014; Fang et al., 2014). It is important to have effective symptom management so patients do not suffer needlessly.

A powerful predictor of suicide ideation and completed suicide is hopelessness (Abbey, Rosenfeld, Pessin, & Breitbart, 2006). Surprisingly, suicide in the general population is most significantly associated with risky behavior, psychomotor agitation, and impulsivity, which can be associated with mixed depression (Harrison, 2015), where the patient is depressed but also has symptoms of excitation. Table 1 lists some of the warning signs of suicide.

**Table 1:**
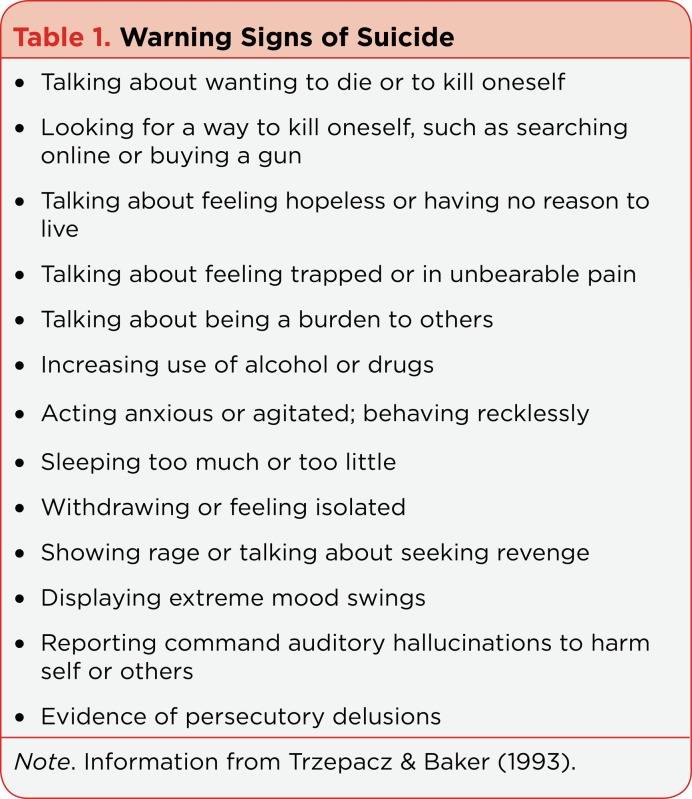
Warning Signs of Suicide

##  Assessment

Assessment for suicide should occur frequently along the cancer continuum. Advanced practitioners are in an ideal position to add this type of screening into their patient encounters. Components should include patient and family histories of suicide, suicidal attempts, psychiatric disorders (especially depression), as well as a history of drug or alcohol use or abuse (Aiello-Laws, 2010). There are no reliable screening tools to help prevent suicide in cancer populations, but there are reliable screening tools to detect depression: the Beck Hopelessness Scale; Diagnostic and Statistical Manual of Mental Disorders, Fifth Edition criteria; Patient Health Questionnaire; Endicott criteria using the Hamilton Depression Rating Scale; and simply asking, "Are you depressed?" (Anguiano et al., 2012). Asking this question will not put the idea into a patient’s head. When asked that question, most patients adamantly deny wanting to harm themselves. If these patients are so distressed to have suicidal thoughts, they affirm these thoughts. Kissane et al. (2004) developed a Demoralization Scale, which can also predict suicidality.

The National Comprehensive Cancer Network (NCCN) introduced the Distress Thermo-meter for use by patients and clinicians. The NCCN (2015) uses the word distress rather than depression to avoid any stigma and to facilitate discussion. The tool uses a visual thermometer, ranging from 0 for no distress and 10 indicating extreme distress; it consists of 36 yes/no questions. Scores of 4 or higher indicate clinically significant depressive symptoms (Ransom, Jacobsen, & Booth-Jones, 2006).

There is a suicide risk assessment tool designed for non–mental health professionals to use for patients (Patterson, Dohn, Bird, & Patterson, 1983). It goes by the acronym SAD PERSONS (Table 2) and assigns 1 point to each of 10 items. A score from 7 to 10 indicates that the person is at high risk for attempting suicide.

**Table 2:**
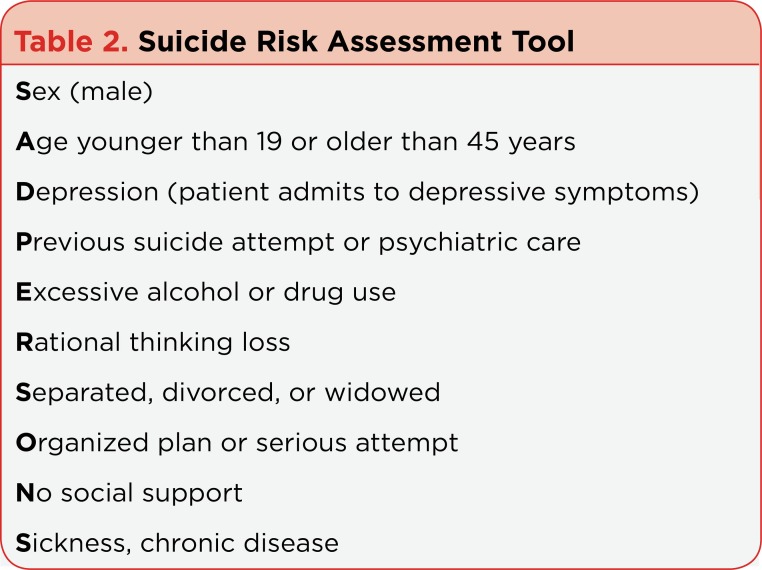
Suicide Risk Assessment Tool

Suicide ideation includes serious thoughts, verbalizations, or behavioral indications about a possible attempted suicide. The source of the information can be the patient or an observer. If there is concern about suicide risk, patients should be asked about their access to lethal means, such as a gun in the house or unused bottles of medications (Aiello-Laws, 2010). If the concern is more immediate, patients should be assessed for a plan and method and the method removed. Other steps include ensuring a safe environment and staying with the patient, either on the phone or in person; as well as assessing social supports and mobilizing as much of their support system as possible and alerting the health-care team. Suicidal remarks, gestures, or self-destructive comments and/or behaviors should be documented in the patient’s medical record.

Suicide precautions include actions implemented, such as patient observation, evaluation of the immediate physical environment, and implementation of physician directives. Suicide precautions should be instituted immediately and patients should be referred to appropriate resources (Valente, 2010; NCCN, 2015). Patients should be provided with a crisis hotline number (Lifeline: 1-800-273-TALK [8255]).

If the patient is in the hospital, call a security officer. Find out whether you need to write an order for one-to-one constant observation for safety. If the patient is in the clinic and there is inadequate staff or assistance to monitor and control the patient’s behavior, call 911. If the patient is at home, ask a family member to bring the patient to the clinic or emergency department. If no one can do so, call 911 to take the patient to the nearest emergency department (Roth & Levenson, 2015). Since this can be a life or death situation, family members can be notified without fear of violating HIPAA (Health Insurance Portability and Accountability Act) legislation. Table 3 offers a list of resources about suicide for advanced practitioners.

**Table 3:**
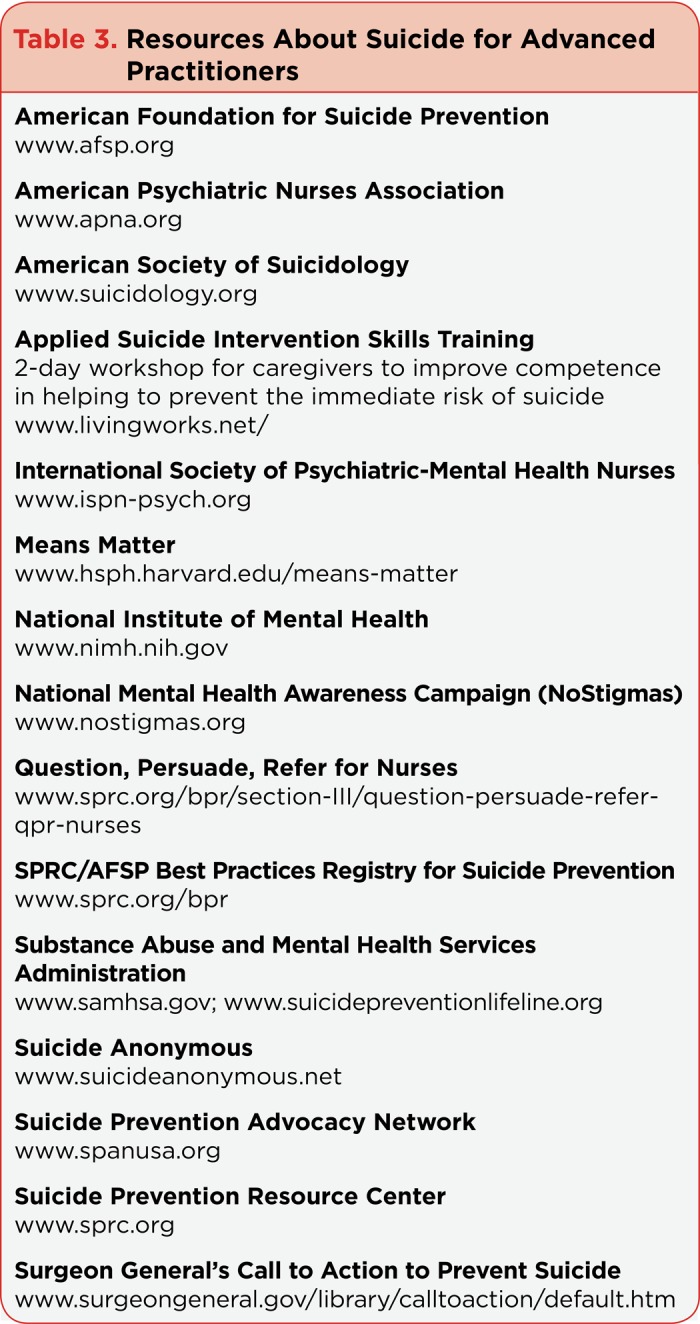
Resources About Suicide for Advanced Practitioners

## Saving Lives Through Prevention 

The goal of suicide prevention is to stop suicide attempts. As advanced practitioners are with patients more than any other health-care worker, their role is vital in preventing suicide. The goal of intervention is to work with patients to improve coping skills, reshape negative thoughts, and mobilize a support system while developing and maintaining a supportive alliance (Aiello-Laws, 2010). According to Chochinov (2001), the most effective intervention for depression is medication combined with psychosocial support and counseling. An apathetic attitude toward suicidality can be lethal. According to Bolster, Holliday, Oneal, and Shaw (2015), as more advanced practitioners are trained in suicide prevention, more lives can be saved.
